# Significant Increase of Erectile Dysfunction in Men With Post-stroke: A Comprehensive Review

**DOI:** 10.3389/fneur.2021.671738

**Published:** 2021-07-28

**Authors:** Shankun Zhao, Weizhou Wu, Panxing Wu, Chao Ding, Bingxiang Xiao, Zhengbao Xu, Yan Hu, Maolei Shen, Lu Feng

**Affiliations:** ^1^Department of Urology, Taizhou Central Hospital (Taizhou University Hospital), Taizhou, China; ^2^Department of Urology, Maoming People's Hospital, Maoming, China; ^3^Department of Neurosurgery, Taizhou Central Hospital (Taizhou University Hospital), Taizhou, China; ^4^Department of Obstetrics and Gynecology, Taizhou Central Hospital (Taizhou University Hospital), Taizhou, China

**Keywords:** stroke, erectile dysfunction, prevalence, comprehensive review, risk

## Abstract

Men with erectile dysfunction (ED) are considered to be at risk from stroke events. Conversely, post-stroke patients are also at high risk of ED, whereas a quantitative result from all the relevant studies has not been previously addressed. Therefore, we have performed a comprehensive review and meta-analysis on this issue. This study was registered on PROSPERO (ID No. CRD42021226618). Twenty studies with a total of 3,382 stroke events were included, of which six studies were included for quantitative analysis, and the remaining 14 studies were calculated for the ratio of ED. Synthetic results from four eligible studies providing the ED cases showed that stroke patients were associated with a significantly higher risk of ED than the general population [pooled relative risk (RR) = 3.32, 95% confidence interval (CI): 1.25–8.82, *P* = 0.016]. Men with stroke were also found to be associated with a significant decline in International Index of Erectile Function −5 (IIEF-5) score as compared with the healthy controls [three studies, standard mean differences (SMD) = −1.8, 95% CI: −2.94 to −0.67, *P* = 0.002]. The prevalence of ED in post-stroke patients among 14 studies ranged from 32.1 to 77.8%, which was dramatically higher than that of the general population. The result of the GRADE-pro revealed that the quality of the evidence in this study was moderate. The present study has confirmed the high prevalence of ED in men with stroke. ED in stroke patients is a result of both neurological and psychological factors. Rehabilitative interventions rather than phosphodiesterase-5 (PDE-5) inhibitors are recommended to improve the erectile function for those survivors with ED.

## Introduction

Stroke is a debilitating cerebrovascular event characterized by impaired blood flow to the brain causing rapid neuron death due to hemorrhage or extremely serious oxygen and energy deprivation. According to the common causes, stroke can be classified into four main types, including ischemic, hemorrhagic, transient ischemic attack, and cryptogenic (1). Ischemic stroke, the most common type of stroke (71% of cases), is mainly caused by a sudden obstruction in the cerebral vessels due to a clot or occlusion, resulting in the blockage of blood supply to a focal region of the brain (2). Hemorrhagic stroke is the second common type of stroke, which is characterized by releasing blood into the brain on the account of the narrowing or abrupt rupturing of cerebrovascular vessels (3). Stroke is considered as a pivotal global health problem due to it is now the third leading cause of death after heart disease and cancer (4). As reported, stroke-inducing deaths have risen globally from 5.3 million in 2007 to 6.2 million in 2017 (5). The number of stroke survivors is expected to increase to over 10 million in 2030 (6).

Stroke also serves as the third most common cause of disability globally (7). Disability is a long-term sequela after stroke. A large sample study indicated that stroke survivors had a significantly higher prevalence of post-stroke disability than the control group with the same comorbidities (32.8 vs. 23.5%) (8). Patients with post-stroke disabilities mainly accompanied by hemiplegic paralysis, aphasia, and dysphagia. Moreover, patients with a history of stroke are vulnerable to some other impairments, including cognitive disabilities, reactions, and emotional changes (9). Of all post-stroke disabilities, sexual dysfunction (SD) is an under-recognized event that should be extensively discussed as other sequelae. SD in post-stroke patients is commonly due to the damage of brain areas controlling sexual behavior (10). In addition, the co-morbidity, general health status, and psychological factors are important contributors to the post-stroke SD (11). The prevalence of SD after stroke ranges from 20 to 95%, occurring in either gender (12, 13). Nasimbera et al. (14) reported that SD in both sexes was present in 75% of post-stroke patients, whereas the prevalence of SD in the healthy controls was only 22.5%. A national population-based study including both genders showed that sexual activity was significantly lower in patients with stroke than in those without stroke (60 vs. 30.8%) (15). Due to stroke occurs more frequently in men than in women (133/10,000 in men and 99/10,000 in women annually) (2), erectile dysfunction (ED) was more commonly reported in men across studies. Ossou-Nguiet et al. (16) indicated that the frequency of ED after stroke was 51.92% (mean age: 56 years). In a more previous study, Korpelainen et al. (17) reported that the prevalence of ED in post-stroke patients was up to 74% (mean age: 59 years). Referring to these two studies conducted in European countries, the ED prevalence in post-stroke patients was dramatically elevated when compared with that of the general population in Europe (19%) (18). Though a significant decline in erectile functioning was observed in men with stroke, some patients might regain erectile capacity after a delay of 7 weeks following the stroke (19). However, a fairly large number of patients did not seem to resume a normal erection and even worsening in time (10, 20).

Stroke leads to ED and *vice versa*. Several meta-analyses (21–23) suggested that ED is also an independent risk factor for stroke development and showed a 34–35% higher risk of stroke in men with ED than in those without ED. Dong et al. (21) showed that ED significantly increased the risk of stroke compared with the reference group [relative risk (RR) = 1.35, 95% confidence interval (CI): 1.19–1.54]. Vlachopoulos et al. (22) demonstrated that the presence of ED might increase the risk for future cardiovascular events, including stroke. Zhao et al. (23) indicated that ED independently predicted and significantly increased the risk of stroke (RR = 1.34, *P* < 0.001).

Currently, however, how risky ED is in post-stroke patients compared with the general population is still unknown. In the present study, we aim to summarize all the evidence on the association between stroke and ED and further calculate a quantitative result by a meta-analysis to facilitate clinical understanding of this issue.

## Materials and Methods

As shown in Supplementary Table 1, this systematic review and meta-analysis was followed by the Preferred Reporting Items for Systematic Reviews and Meta-Analyses (PRISMA) guidelines. This study was registered on PROSPERO (ID No. CRD42021226618).

### Data Sources and Search Strategy

Four common databases, including MEDLINE (PubMed), EMBASE (OVID), Cochrane Library, and PsycINFO, were systematically searched to filter the eligible studies prior to December 1, 2020. We only included those studies reporting with English language and investigating with human participants. The search strategy used for detecting the potential studies in PubMed databases was: (((((((((((((((((((((((((((((“Stroke”[Mesh]) OR (Strokes)) OR (Cerebrovascular Accident)) OR (Cerebrovascular Accidents)) OR (CVA (Cerebrovascular Accident))) OR (CVAs (Cerebrovascular Accident))) OR (Cerebrovascular Apoplexy)) OR (Apoplexy, Cerebrovascular)) OR (Vascular Accident, Brain)) OR (Brain Vascular Accident)) OR (Brain Vascular Accidents)) OR (Vascular Accidents, Brain)) OR (Cerebrovascular Stroke)) OR (Cerebrovascular Strokes)) OR (Stroke, Cerebrovascular)) OR (Strokes, Cerebrovascular)) OR (Apoplexy)) OR (Cerebral Stroke)) OR (Cerebral Strokes)) OR (Stroke, Cerebral)) OR (Strokes, Cerebral)) OR (Stroke, Acute)) OR (Acute Stroke)) OR (Acute Strokes)) OR (Strokes, Acute)) OR (Cerebrovascular Accident, Acute)) OR (Acute Cerebrovascular Accident)) OR (Acute Cerebrovascular Accidents)) OR (Cerebrovascular Accidents, Acute)) AND ((((((“Erectile Dysfunction”[Mesh]) OR sexual function) OR sexual dysfunction) OR “Sexual Dysfunctions, Psychological”[Mesh]) OR “Sexual Dysfunction, Physiological”[Mesh]) OR Impotence). Furthermore, we attempted to identify additional studies by manual inspection of reference lists in the relevant articles.

### Assessments of Stroke and ED

Both of the definitions of stroke and ED were based on the World Health Organization (WHO) and the International Classification of Diseases (ICDs) codes. The diagnosis of stroke was commonly confirmed by computerized tomography (CT) and/or magnetic resonance imaging (MRI). We accepted all types of stroke, including ischemic, hemorrhagic, transient ischemic attack, and cryptogenic stroke. ED is diagnosed with any of the validated tools as reported in the eligible studies, such as the International Index of Erectile Function −5 (IIEF-5), ICD, enquired by the clinicians, questionnaires, and prescription for ED.

### Study Selection

Any studies reporting the prevalence of ED in post-stroke patients with or without a control group were considered eligible. The inclusion criteria for this systematic review and meta-analysis were followed with the standard of Patient, Intervention, Comparison, Outcome, and Study design (PICOS). The question guiding for this study was: is there a positive association between stroke and ED? The components for the PICOS evidence were: adult men with ED or impotence (P), a history of stroke (I), compared with the normal healthy subjects^©^, the confirmation of ED (O), and any study designs (S). In addition, those studies representing with the RR, hazard ratio (HR), or odds ratio (OR) with 95% CI or sufficient data to calculate the effect sizes were included. Besides, in this study, we further included those relevant studies without providing the information of the control group due to most of the related studies investigating the relationship between stroke and ED only focused on the crude prevalence of ED in stroke patients but lack of the ED prevalence in a control group with similar comorbidities in clinical practice. Since this is a comprehensive review, including all relevant studies could better facilitate clinical understanding of the prevalence of ED in post-stroke patients among different countries and samples. The exclusion criteria in this study were: (1) review articles, editorial letters, comments for published studies, and case reports; (2) duplicated data or previous publications for the same samples and the scientific questions; and (3) animal or *in vitro* experiments. The process of study selection was performed by two authors independently. Any ambiguities were resolved through a third author or the corresponding author.

### Data Extraction

The following information was extracted according to a conventional data collection form, including the first authors' names of the included studies, publication year, country, study design, age of the participants, ED cases (ratio), assessment and type/classification of stroke, assessment of ED, and scores of IIEF-5 with mean and standard deviation.

### Quality Assessment

The methodological quality of the included cross-sectional studies was dependent on the gained scores from the cross-sectional study quality methodology checklist: 0–3 (low quality), 4–7 (moderate quality), and 8–11 (high quality). While the methodological quality of cohort and case–control studies was followed by the Newcastle–Ottawa Scale (NOS), low quality, moderate quality, and high quality have corresponded to scores of 0–3, 4–6, and 7–9, respectively. To further rank the overall quality of the evidence from this meta-analysis, the evaluation of the Grading of Recommendations Assessment, Development, and Evaluation (GRADE) approach was employed.

### Risk of Bias Assessment

The risk of bias assessments for the included studies was conducted by using the software Review Manager 5.3 (Nordic Cochrane Centre, Cochrane Collaboration, Copenhagen, Denmark).

### Meta-Analyses

All analyses were completed using STATA version 13.0 software for Windows (StataCorp LP, College Station, TX, USA). In this study, the overall RR with its 95% CI synthesized by all the eligible studies was used to quantitatively estimate the strength of the association between stroke and ED. In addition, the standard mean differences (SMD) and the corresponding 95% CI were applied to calculate the combined effects for the continuous variables (i.e., IIEF-5 scores). Statistical significance was assumed at a two-tailed *P* < 0.05. *I*^2^ statistic and Cochrane *Q* statistic were used for the heterogeneity test. Of which, *I*^2^ >50% was considered as substantial heterogeneity, and the *P*-value of the *Q* test <0.10 was supposed to be statistically significant. A random-effect model rather than a fixed-effects model was applied in this study due to a high likelihood of between-study variance for differences in both studies designed and demographic characteristics. Moreover, sensitivity analyses were conducted to find the potential origin of the between-study heterogeneity. Finally, the funnel plot, Begg's rank correlation test, and Egger's regression asymmetry test were used to identifying the publication bias.

## Results

### Literature Search

**Figure 1:**
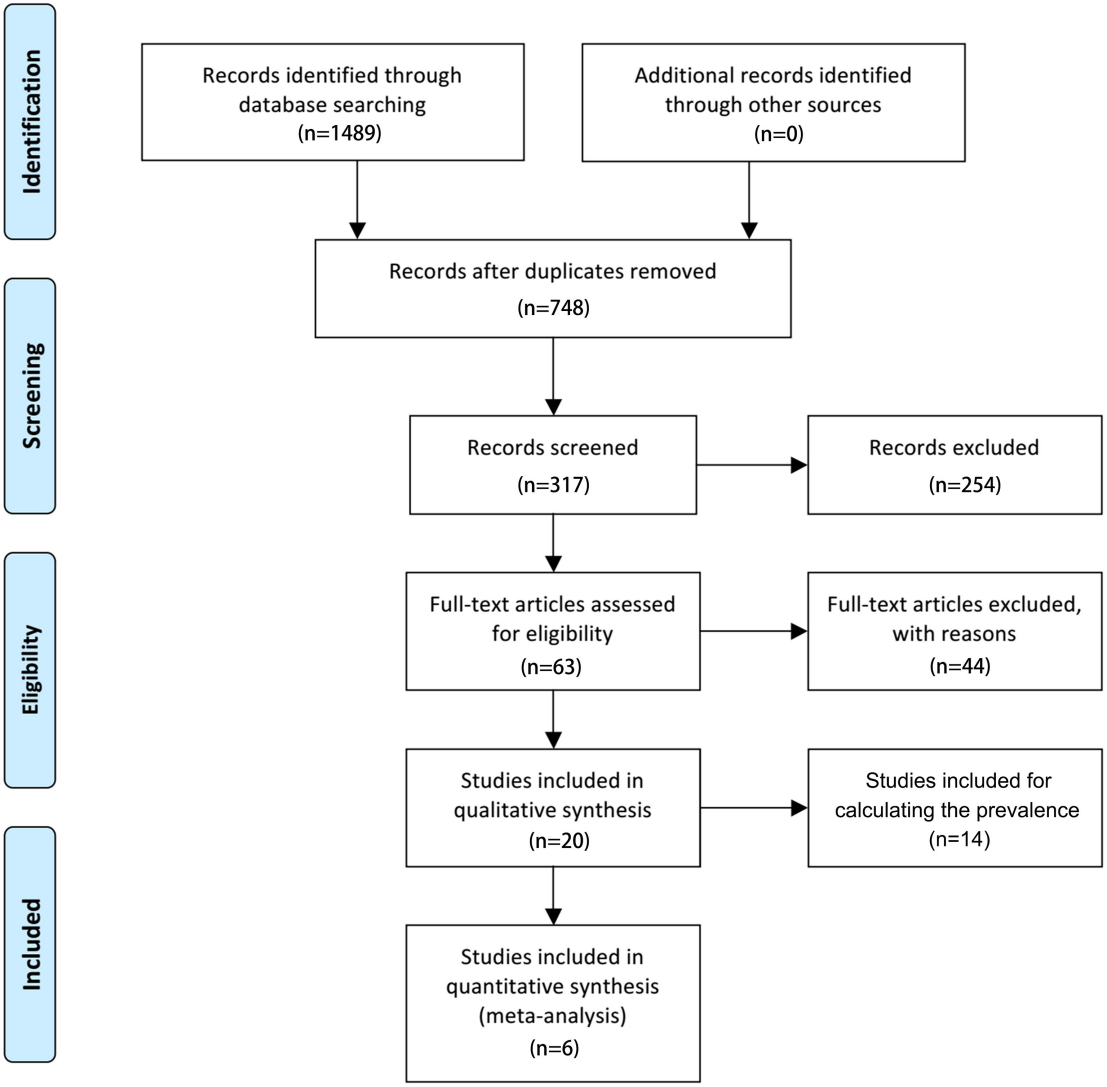
Flowchart of the study selection.

The search flowchart for screening the relevant studies is shown in Figure 1. During the initial database search, 1,489 publications were identified, of which 717 were from MEDLINE, 356 from EMBASE, 189 from Cochrane Library, and 227 from PsycINFO database. Finally, six eligible studies (24–29) with a total of 11,552 participants (1,244 individuals were stroke patients) met the predefined inclusion criteria for calculating the overall RR or SMD through a meta-analysis. Among the six included studies, four studies (24, 26, 27, 29) provided the number of ED cases that could be synthesized for the RR with 95% CI by these dichotomous variables, two studies (24, 26) provided the IIEF-5 scores that were calculated for the pooled SMD by these continuous variables, and 1 study (26) provided both the number of ED cases and IIEF-5 scores.

As aforementioned, we further included those studies that only provided the ED cases in the stroke sample but without a healthy control group because we want to show the crude ED prevalence among stroke patients for different countries or races. After exhaustive searching, we have identified 14 eligible studies (12, 17, 20, 30–40) that reported the ratio of ED in stroke subjects. Non-English clinical trials were excluded from this study. The trial publication years of the eligible studies ranged from 1998 to 2019. Except for three studies represented with a broad age group of the population, the mean age of the individuals in the remaining studies ranged from 45 ± 12 to 64.93 ± 8.81 years. Of the 20 included studies, 12 trials were conducted in Europe, six trials in Asia, and two trials in Africa. Assessments for stroke commonly used CT and/or MRI. Types of stroke among these included studies were mainly ischemic and/or hemorrhagic. There were various evaluations for ED, including enquired, ICD, IIEF-5, and specific questionnaire. The characteristics of the six included studies for meta-analysis and 14 included studies for calculating the ED prevalence are summarized in Table 1.

**Table 1:** Characteristics of the 20 included studies.

**Study**	**Study design**	**Mean age (years)**	**Study group case (ED)/total**	**Control group case (ED)/total**	**IIEF-5: mean ± SD**	**Assessment and type of stroke**	**Assessment of ED**
**Four included studies that provided the data from both the study group and the control group that could be calculated for the pooled RR** ***via*** **a meta-analysis**							
Parazzini et al. (27) Italy	Cross-sectional	18 over 70	15/25	242/1,985	NA	Assessment: evaluation was not available Classification: thrombotic/hemorrhagic	Enquired
Chung et al. (24) Chinese Taipei	Cohort	S and C: 58.5 ± 11.4	188/918	1,313/8,088	NA	Assessment: ICD-9-CM codes 430–438, further validation by CT or MRI Classification: NA	ICD-9-CM code 607.84; ICD-9-CM code 302.72
Koehn et al. (26) Germany	Case–control	S: 62.6 ± 10.5 C: 61.7 ± 11.2	45/57	6/22	S: 13.1 ± 8.9 C: 21.8 ± 4.5	Assessment: cranial CT or MRI Classification: ischemic stroke	IIEF-5
Tibaek et al. (29) Denmark	Cross-sectional	s S: 67–79 C: 59–66	8/30	1/64	NA	Assessment: confirmed by CT or MRI scans Classification: without specific description s	IIEF-5
**Two included studies that provided the IIEF-5 score that could be calculated for the pooled SMD** ***via*** **a meta-analysis**							
Jung et al. (25) Korea	Case–control	S and C: 64.93 ± 8.81	Total: 109	Total: 109	S: 5.89 ± 7.08 C: 10.67 ± 7.1	Assessment: CT or MRI scan Classification: Ischemic stroke and hemorrhagic stroke	IIEF-5
Sikiru et al. (28) Nigeria	Case–control	S and C: 50–78	RH: 55; LH: 50	Total: 40	RH: 7.55 ± 4.07 LH: 10.40 ± 5.7 C: 21.5 ± 4.27	Assessment: CT or MRI scan Classification: without specific description	IIEF-5
**Fourteen included studies that only provided the data from the study group but not for the control group, which could not be calculated for the pooled RR** ***via*** **a meta-analysis (several published studies were excluded due to a Non-english language)**							
Korpelainen et al. (20) Finland	NA	S: 53.5 ± 8.2	19/38 (50%)	NA	NA	Assessment: Scandinavian Stroke Scale Classification: brain infarction and intracerebral hemorrhage	Questionnaires
Korpelainen et al. (17) Finland	Cross-sectional	S: 59.1 ± 10.2	87/117 (74.4%)	NA	NA	Assessment: NA Classification: brain infarction and hemorrhagic stroke	Enquired
Cheung et al. (32) China	Cross-sectional	S: 54.0 ± 11.0	37/62 (59.7%)	NA	NA	Assessment: NA Classification: ischemic and hemorrhagic stroke	Enquired
Choi-Kwon and Kim (27) Korea	Cross-sectional	S: mean: 56	18/56 (32.1%)	NA	NA	Assessment: CT/MRI Classification: infarcts and hemorrhages	NA
Ponholzer et al. (36) Austria	Cohort	S: 61.3 ± 5.3	294/644 (45.7%)	NA	S: 18.9 ± 5.4	Assessment: medical history Classification: without specific description	IIEF-5
Bener et al. (30) UK	NA	S: 56.1 ± 9.8	292/605 (48.3%)	NA	NA	Assessment: NA Classification: subarachnoid hemorrhage, intracranial hemorrhage, and cerebral infarction	IIEF-5
Tamam et al. (39) Turkey	Cohort	S: 58.0 ± 9.8	40/63 (63.5%)	NA	NA	Assessment: National Institutes of Health Stroke Scale Classification: without specific description	Enquired
Ponholzer et al. (37) Austria	Cohort	S: 45 ± 12	8/16 (50%)	NA	NA	Assessment: NA Classification: NA	IIEF-5
Akinpelu et al. (12) Nigeria	Cross-sectional	S: 57.0 ± 10.0	37/60 (61.7%)	NA	NA	Assessment: NA Classification: without specific description	NA
Bugnicourt et al. (31) France	Cohort	S: 48.0 ± 9.4	8/17 (47.1%)	NA	NA	Assessment: MRI and/or angiography Classification: ischemic stroke	NA
Epprecht et al. (35) Switzerland	NA	S: 53.75 ± 8.88	7/14 (50%)	NA	NA	Assessment: CT scan Classification: subarachnoid hemorrhage stroke	IIEF
Winder et al. (40) Germany	Cross-sectional	S: 60.5 ± 10.5	32/52 (61.5%)	NA	NA	Assessment: CT/MRI Classification: ischemic stroke	IIEF-5
Dai et al. (34) China	Cross-sectional	S: 55.58 ± 6.3	249/320 (77.8%)	NA	S: 16.36 ± 5.57	Assessment: MRI Classification: ischemic stroke	IIEF-5
Purwata et al. (29) Indonesia	Cross-sectional	S: 52.19 ± 4.37	38/74 (51.4%)	NA	S: 17.82 ± 6.56	Assessment: patients visited the neurology polyclinic Classification: ischemic stroke and hemorrhagic stroke	IIEF-5

### Study Quality and Quality of the Evidence

As shown in Supplementary Tables 2, 3, across the six eligible studies for meta-analysis, four studies (25–28) were assumed as moderate methodological quality, and two studies (24, 29) were regarded as high quality. The proportion of high methodological quality study was 33.3% (2/6).

To rank the quality of the evidence from this meta-analysis, the GRADE approach was applied (Table 2). Results from GRADE-pro generated by the 4 included studies (24, 26, 27, 29) providing the ED cases indicated that the rate of events of ED on average in patients with stroke was 256/1,030 (24.9%), whereas the rate of ED in the general population was 1,562/10,159 (15.4%). The absolute effect of stroke on ED was 357 more per 1,000 (from 38 more to 1,000 more). The overall quality of the evidence was ranked as moderate.

**Table 2:** GRADE-profiler summary of evidence for the effects of stroke and erectile dysfunction.

**Quality assessment**							**No. of patients**		**Effect**		**Quality**	**Importance**
**No. of studies**	**Design**	**Risk of bias**	**Inconsistency**	**Indirectness**	**Imprecision**	**Other considerations**	**Hyperthyroidism**	**Control**	**Relative** **(95% CI)**	**Absolute**		
**Erectile dysfunction [assessed with: IIEF-5, International Classification of Disease (ICD), or enquired]**												
4	Observational studies	Serious^a^	No serious inconsistency^b^	No serious indirection^c^	No serious imprecision	Very strong association^d^	256/1,030 (24.9%)	1,562/10,159 (15.4%)	RR = 3.32 (1.25–8.82)	357 more per 1,000 (from 38 more to 1,000 more)	⊕⊕⊕○ Moderate	Critical

*CI, confidence interval; RR, relative risk. GRADE Working Group grades of evidence: High quality: further research is very unlikely to change our confidence in the estimate of effect. Moderate quality: further research is likely to have an important impact on our confidence in the estimate of effect and may change the estimate. Low quality: further research is very likely to have an important impact on our confidence in the estimate of effect and is likely to change the estimate. Very low quality: we are very uncertain about the estimate*.

a *Selection bias, performance bias, and detection bias were identified in some of these included studies*.

b *Forest plots of the meta-analysis revealed that all the four included studies yielded a significant association between stroke and erectile dysfunction*.

c *The mechanisms of stroke-induced erectile dysfunction were mainly dependent on the damage of brain areas controlling sexual behavior, combining with the co-morbidity, general health status, and psychological factors*.

d *Very large sample, a total of 11,189 participants were included from four included studies; the synthetic RR showed that patients with stroke were 3.32 times more likely to suffer from erectile dysfunction than the healthy controls*.

The results of the risk of bias table and graph are illustrated in Supplementary Figure 1; all the included studies were considered as “high risk” on account that all of them were retrospectively designed.

### Meta-Analysis and the Crude Prevalence of ED

**Figure 2:**
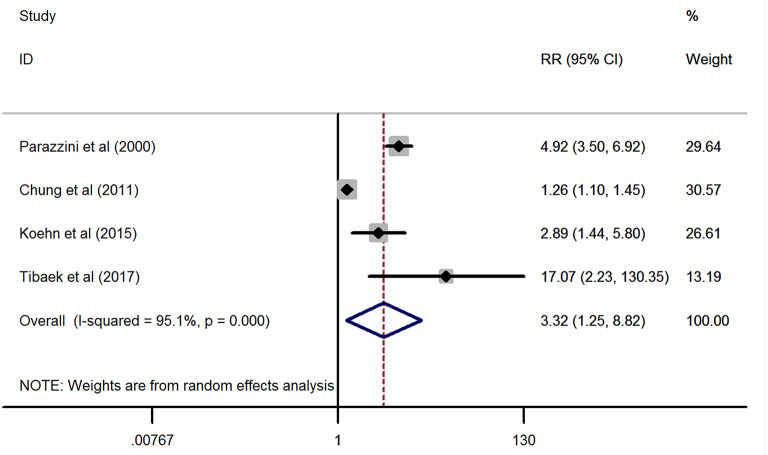
Forest plots of the meta-analysis from the four eligible studies on the association between stroke and erectile dysfunction.

As displayed in Figure 2, the combined effect from four included studies (24, 26, 27, 29) providing the ED cases supported the significant positive association between stroke and ED (pooled RR = 3.32, 95% CI: 1.25–8.82, *P* = 0.016) by using a random-effects model. However, statistical heterogeneity was detected during this analysis (*I*^2^ = 95.1%, *P* < 0.001).

**Figure 3:**
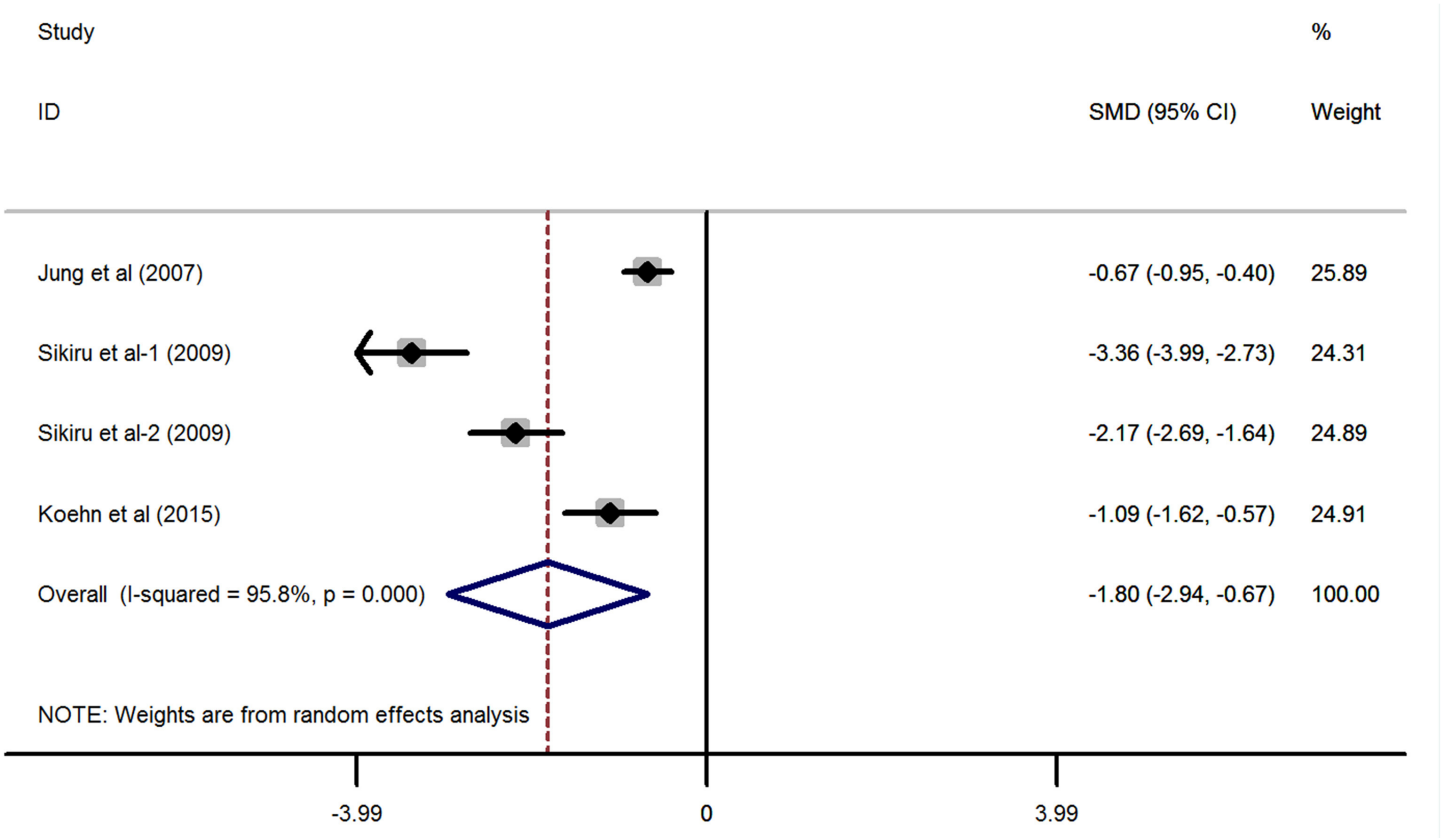
Forest plots of the meta-analysis based on the three included studies reporting the IIEF-5 scores.

Referring to Figure 3, in line with the above finding, the synthetic effect of the continuous variables derived from three studies (25, 26, 28) providing the IIEF-5 scores indicated that adult patients with stroke had a significantly lower value of IIEF-5 scores than the general population without a stroke (SMD = −1.8, 95% CI: −2.94 to −0.67, *P* = 0.002; heterogeneity: *I*^2^ = 95.8%, *P* < 0.001). Of note, the study by Sikiru et al. (28) has provided both the IIEF-5 scores from the right hemiplegia and left hemiplegia impairments, thus setting as Sikiru-1 (right hemiplegia) and Sikiru-2 (left hemiplegia) in the meta-analysis.

**Figure 4:**
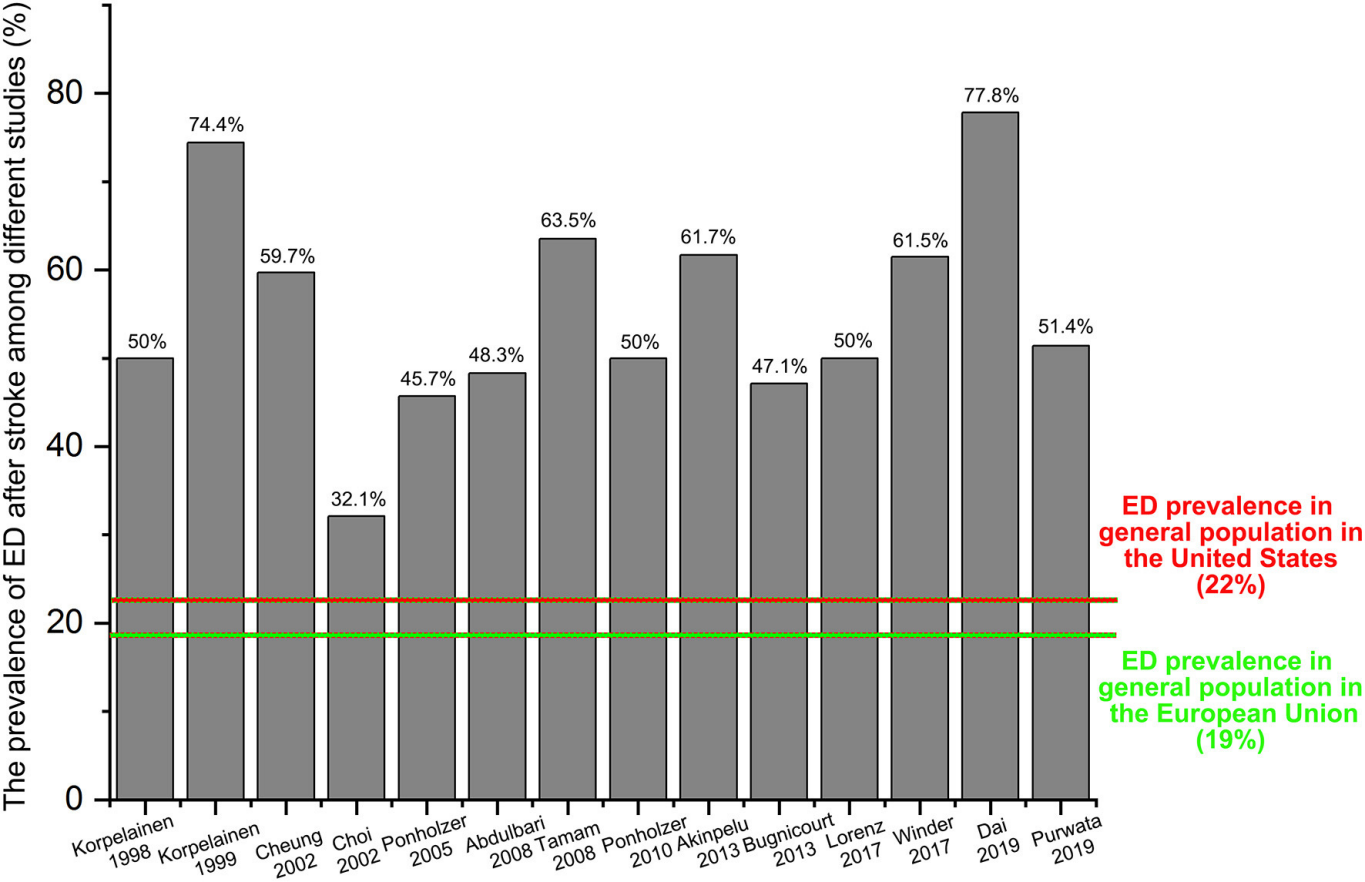
Crude ED prevalence of the 14 included studies.

To better illustrate the ratio of ED in stroke patients, we have collected all the data from the relevant clinical studies through a comprehensive review. Finally, 14 studies (12, 17, 20, 30–40) without a control group have been identified, and most of these studies were conducted in Europe (nine studies: 64.3%). As shown in Figure 4, the prevalence of ED among these studies ranged from 32.1 to 77.8% that was dramatically higher than that of the general population originated from the ED epidemiological investigations of the European Union (ED prevalence: 19%, marked with a green color) or the United States (ED prevalence: 22%, marked with a red color).

### Sensitivity Analysis

To evaluate the influence of an independent study on the overall RR and heterogeneity derived from four studies (24, 26, 27, 29) providing the ED cases, we further performed a sensitivity analysis (Supplementary Figure 2 and Table 3). The results showed that the overall pooled RR raising to 4.46 (95% CI: 2.58–7.7, *P* < 0.001) and the substantial heterogeneity were eliminated after omitting the study developed by Chung et al. (24) (*I*^2^ = 42%, *P* < 0.001). On the other hand, sensitivity analysis also indicated that there was no significant association between stroke and ED after omitting the studies by Parazzini et al. (27) (pooled RR = 2.61, 95% CI: 0.97–7.05, *P* = 0.058; heterogeneity: *I*^2^ = 82.5%, *P* = 0.003) or Tibaek et al. (29) (pooled RR = 2.59, 95% CI: 0.93–7.19, *P* = 0.068; heterogeneity: *I*^2^ = 96.4%, *P* < 0.001). These results revealed that there were some variations in the combined RR and heterogeneity after omitting one of the included studies. The main reason for this inconsistency was that only four studies were included and the sample size was distinctly different among each study.

**Table 3:** Sensitivity analysis after each study was excluded by turns.

**Study omitted**	**RR with 95% CI for remainders**	**Heterogeneity**	
		***I*** ^**2**^	***P***
Parazzini et al. (27)	2.61 (0.97–7.05), *P* = 0.058	82.5%	0.003
Chung et al. (24)	4.46 (2.58–7.7), *P* < 0.001	42%	0.178
Koehn et al. (26)	3.59 (1.05–12.28), *P* = 0.042	96.6%	<0.001
Tibaek et al. (29)	2.59 (0.93–7.19), *P* = 0.068	96.4%	<0.001

*RR, relative risk; CI, confidence interval*.

### Publication Bias

As shown in Supplementary Figure 3, results from the funnel plots revealed that no significant publication bias was observed among the four eligible studies for pooling RR through a meta-analysis (Begg's, *P* > |z| = 0.734; Egger, *P* > |t| = 0.267, 95% CI: −8.03–16.83).

## Discussion

Sexual health is an integral part of the quality of life. Various extragenitourinary diseases have been confirmed to contribute to the impairment of sexual life, e.g., diabetes mellitus, cardiovascular disease, dyslipidemia, and some digestive diseases. In 1981, Bray et al. (41) were the first to be conscious that stroke might induce SD in both sexes. Many investigators realize that sexuality is an important issue in post-stroke rehabilitation, thus mounting clinical studies have been conducted to better illustrate the association between stroke and sexual functioning (15, 16). Oni et al. (42) reported that stroke survivors regardless of gender were more likely to develop SD than the controls (16/70, 22.9% vs. 4/70, 5.7%). Yilmaz et al. (43) concentrated on the sexual function of female stroke survivors and observed that the scores of total Female Sexual Function Inventory (FSFI) and FSFI subgroups in patients with stroke were significantly lower than those in healthy controls. Since stroke is more frequent in males than in females, SD is more common among male stroke patients. Duits et al. (44) included 19 male stroke patients and found that their orgasmic function and sexual desire were remarkably lower. Monga et al. (45) suggested that men might suffer from a significant ejaculatory problem in the period after stroke. In clinical practice, there are more trials focused on the changes in erectile function in stroke patients than in orgasm, sexual desire, or ejaculation disorder.

According to the four included studies providing the ED cases, synthetic results revealed that adult males with stroke were at 3.32-fold higher risk of ED than the general population without stroke (RR = 3.32, 95% CI: 1.25–8.82, *P* = 0.016). Ascertained to the GRADE evaluation, the average rates of ED events were 256/1,030 (24.9%) in subjects with stroke and 1,562/10,159 (15.4%) in the healthy controls without stroke. Furthermore, the quality of evidence of this analysis was judged to be moderate. In keeping with the RR calculated from the dichotomous variables, the combined SMD from three eligible studies have suggested that men with stroke have 1.8 scores less than the healthy control subjects and this gap has achieved statistical significance (SMD = −1.8, 95% CI: −2.94 to −0.67, *P* = 0.002). Based on the 14 different clinical trials providing the ED cases in post-stroke patients but lacking a control group, the results showed that the crude prevalence of ED in all these studies (32.1–77.8%) was obviously higher than that of the general population in Europe (19%) or the United States (22%). The methodological quality of the six eligible studies for meta-analysis was moderate to high. Besides, no significant publication bias was identified among these studies. Based on the combined RR, SMD, and crude prevalence of ED in this study, all these results were inclined to confirm that stroke might serve as a risk factor for ED development. Stroke affects erectile functioning and *vice versa*. Several studies have revealed that men with ED were at high risk of stroke (24, 46). Bohm et al. (47) found that ED might be a potent predictor of the composite of stroke in men with cardiovascular disease. A previous meta-analysis showed that the pooled RR of any stroke was 1.35 (95% CI: 1.18–1.53) for ED vs. no ED (48). Thus, ED may be an early warning sign of stroke events. According to the above evidence from our study and the relevant studies, a significant positive association between stroke and ED was verified.

ED after stroke is considered to be correlated with multiple etiologies, including both physical (i.e., hemiparesis, paralysis, and/or dysphasia) and psychosocial causes (i.e., cognitive changes and emotional reactions). Stroke-induced ED was mainly dependent upon impairment of brain areas controlling penile erection or governing sexual behavior. Erections can be modulated by the medial preoptic area, paraventricular nucleus, and anterior hypothalamic regions in the brain. Erectile function is maintained by the autonomic nervous system that controls the interaction of the corpora cavernosa smooth fibers and endothelial cells. The neural pathways for erection include the central nervous system (CNS)-driven sexual behavior, the CNS control of the autonomic system, and the activity of the peripheral nerve (10). The neural impulses are responsible for the integrity of the vascular supply in the penile chambers. In the case of post-stroke patients, impairment of specific brain areas may induce a disruption in central autonomic network structures and pathways that contribute to the erection (10). In addition, it was suggested that autonomic system dysfunction after stroke may contribute to sexual impairment in the affected patients. Of note, different stroke lesions may result in varying frequencies and severities of ED. For example, Koehn et al. (26) indicated that patients with cerebellum stroke suffered less frequent ED (40%) than those with other stroke locations, i.e., middle cerebral artery territory (MCA, 78.8%), brain stem (80%), and posterior cerebral artery territory (80%). It was suggested that stroke lesions in the right cerebellum might be associated with ejaculation disorder (25), whereas MCA territory was responsible for supplying the blood for both the medial frontal lobe and the inferior frontal lobe that were thought to contribute to erectile function (26). Therefore, ED might be more often in patients with MCA stroke, whereas ejaculatory problems seemed to be more common in those with right cerebellum lesions. On the other hand, Koehn et al. (26) also found that a higher prevalence of ED was observed in the right hemisphere stroke than in the left hemisphere lesion (87.5 vs. 70.6%). In line with this finding, previous studies (49, 50) also indicated that stroke patients with right hemisphere damage were more frequently associated with male SD than those with left hemispheric stroke. Jung et al. (25) showed that men with brain lesions in the right pons were linked to a remarkable decrease in the IIEF-5 score. It was demonstrated that the right hemisphere might play a dominant role in the activation/attention of erectile functionality (13). Besides, the right hemisphere is also believed to be dominant in sensing emotional stimuli, and its lesions may increase the susceptibility to an emotional problem that is a key factor for SD development (13). However, a previous study (38) reported a diverse outcome, showing that the prevalence of ED was more frequent in stroke men with left hemisphere lesions than in those with right hemisphere lesions (33.8 vs. 17.6%). Sikiru et al. (28) suggested that lesions in the left basal ganglia affecting libido might in turn influence the right hemiplegic erectile functioning. As a result, stroke location might help to identify those stroke patients at greater ED risk. However, it is worth noting that some investigators even failed to find a positive association between IIEF-5 scores after stroke and stroke location (25).

Brain damage for stroke has long been recognized as the dominating cause of ED development. However, in addition to CNS impairment, co-morbidity and psychological elements might also play roles in post-stroke erectile functions. Mobility restrictions (i.e., hemiplegia or hemiparesis) and spasticity after stroke might cause coital position to be cumbersome (28) or poor coping skills for the affected men, which could compromise their partnership (20). Hemiplegia might limit appropriate body position and movement, whereas spasticity might cause unpredictable dystonic movements and painful muscle spasms during intercourse, all of which could contribute to ED (13). Besides, some treatments for spasticity, such as intrathecal baclofen, may aggravate ED symptoms (51). On the other hand, dominant hemisphere lesions may cause verbal deficits, resulting in depression and diminished self-esteem and confidence in post-stroke patients (20). A Korean study (52) showed that 78% of post-stroke patients had suffered from depression. Both depression and antidepressants are proven to serve as risk factors for ED or deteriorating erectile function (53, 54). Fear of ED was one of the reasons for ceasing sexual activity in men with stroke (20). In addition, fear of a new stroke or rejection by the spouse may contribute to impair the patient's sexual life. Other common sequelae (i.e., fatigue, insomnia, or cognitive deficits), physical impairments (i.e., muscular atrophy, urinary tract symptoms, or bowel dysfunction), and the medications used for preventing further cerebrovascular and cardiovascular events (i.e., antihypertensive agents, beta-blockers, potassium-sparing diuretics, and antipsychotics) after stroke might also be factors worth considering (26, 51, 55).

ED is highly prevalent in men after stroke, and it is an integral part of the quality of life for the survivors. A systematic evaluation and targeted physical examination should be performed when the post-stroke man's presenting concern is ED. More importantly, post-stroke patients with physical and psychological difficulties need to be taken into account in rehabilitation programs, including sexual counseling and pharmacological and rehabilitative interventions. It was suggested that the knowledge possessed by post-stroke patients and their spouses on sexual function was directly associated with sexual frequency and satisfaction (56). Psychosocial elements might be the more important factors influencing post-stroke sexuality rather than neurological deficits. Therefore, psychosocial barriers should be addressed in both patients after stroke and their spouses (57). Since medications used for managing stroke may contribute to ED, stroke survivors are encouraged to discuss alternative medications with their doctors. Phosphodiesterase-5 (PDE-5) inhibitors are the common medications used for ED. However, post-stroke patients might be at elevated risk of hemodynamic impairment after the use of sildenafil citrate (58). In line with this finding, Lorberboym et al. (59) reported that tadalafil administration after stroke might be correlated with decreased blood flow to areas adjacent to the stroke. A comfortable position for intercourse is recommended to improve sexual life for stroke men with hemiparesis and/or spasticity as well as their partners (51). In other studies, Tibaek et al. (60) showed that pelvic floor muscle training might be beneficial to stroke men with ED. A randomized controlled trial developed by Li et al. (61) showed that acupuncture was a safe and effective treatment for post-stroke ED.

Based on the published data of the different clinical characteristics of the post-stroke patients with ED and the non-ED stroke patients, in the risk stratification of patients before the stroke event, individuals with psychosocial disorders (i.e., depression and anxiety), medication use (i.e., antihypertensive drug), comorbidities (i.e., diabetes, hypertension, and hypercholesterolemia), and co-existing medical conditions (i.e., older age, smoking, and obesity) were more vulnerable to suffer from ED than those without these risk factors. In addition to these clinical characteristics, the endothelial dysfunction markers, endothelial progenitor cells (EPCs), and endothelial microparticles are considered to serve as the serum biomarkers for both ED (62) and stroke events (63). Since there is a close association between stroke and ED, either from stroke to ED or from ED to stroke, the low level of EPCs and endothelial microparticles may help to predict those patients with ED at high risk of stroke as well as those stroke patients at high risk of ED.

To our knowledge, this is the first study to demonstrate a significant association between stroke and ED *via* a comprehensive review and meta-analysis. However, some inherent limitations of the study should be noted during the interpretation in clinical practice. First, 20 clinical trials were included in this study, but only six studies provided both the study and control groups that could be used for pooling RR or SMD *via* a meta-analysis. The strength of this study is that all the studies reporting the ED prevalence in stroke samples are included and further analyzed. Referring to the present study, we can not only figure out how risky ED is in post-stroke patients as compared with the general population but also know the crude prevalence of ED in stroke men among different studies. Second, a statistical heterogeneity (*I*^2^ = 95.1%) existed in this meta-analysis. A diverse study design; sample size; age of the participants; countries, types, and locations of the stroke; stroke duration and severity; measurements of ED; and comorbidities could be partly responsible for such heterogeneity.

Results from the pooled RR, SMD, and crude prevalence of ED in stroke patients demonstrated that there is a significant positive association between stroke and ED. Neurological profiles, physical factors, and psychological elements are the important determinants of post-stroke ED. Rehabilitative interventions, psychological therapies, and sexual counseling rather than PDE-5 inhibitors are recommended to improve sexual life or erectile functioning for stroke patients with ED.

## Data Availability Statement

The raw data supporting the conclusions of this article will be made available by the authors, without undue reservation.

## Author Contributions

SZ and LF: conceptualization. WW, PW, CD, and MS: methodology. BX, ZX, and YH: investigation. SZ, PW, and CD: writing—original draft. SZ: writing—review and editing. LF and MS: supervision. All authors contributed to the article and approved the submitted version.

## Conflict of Interest

The authors declare that the research was conducted in the absence of any commercial or financial relationships that could be construed as a potential conflict of interest.

## Publisher's Note

All claims expressed in this article are solely those of the authors and do not necessarily represent those of their affiliated organizations, or those of the publisher, the editors and the reviewers. Any product that may be evaluated in this article, or claim that may be made by its manufacturer, is not guaranteed or endorsed by the publisher.

## Supplementary Material

Supplementary Figure 1 Results of risk of bias assessment in each included study: **(A)** risk of bias graph and **(B)** risk of bias summary.

Supplementary Figure 2 Forest plot for sensitivity analysis.

Supplementary Figure 3 Begg's and Egger's tests to detect publication bias.
