# Supplementary figures and images for: Significant Increase of Erectile Dysfunction in Men With Post-stroke: A Comprehensive Review
Source: Front Neurol. 2021 Jul 28;12:671738. doi: 10.3389/fneur.2021.671738 (PMC8355431; doi:10.3389/fneur.2021.671738)

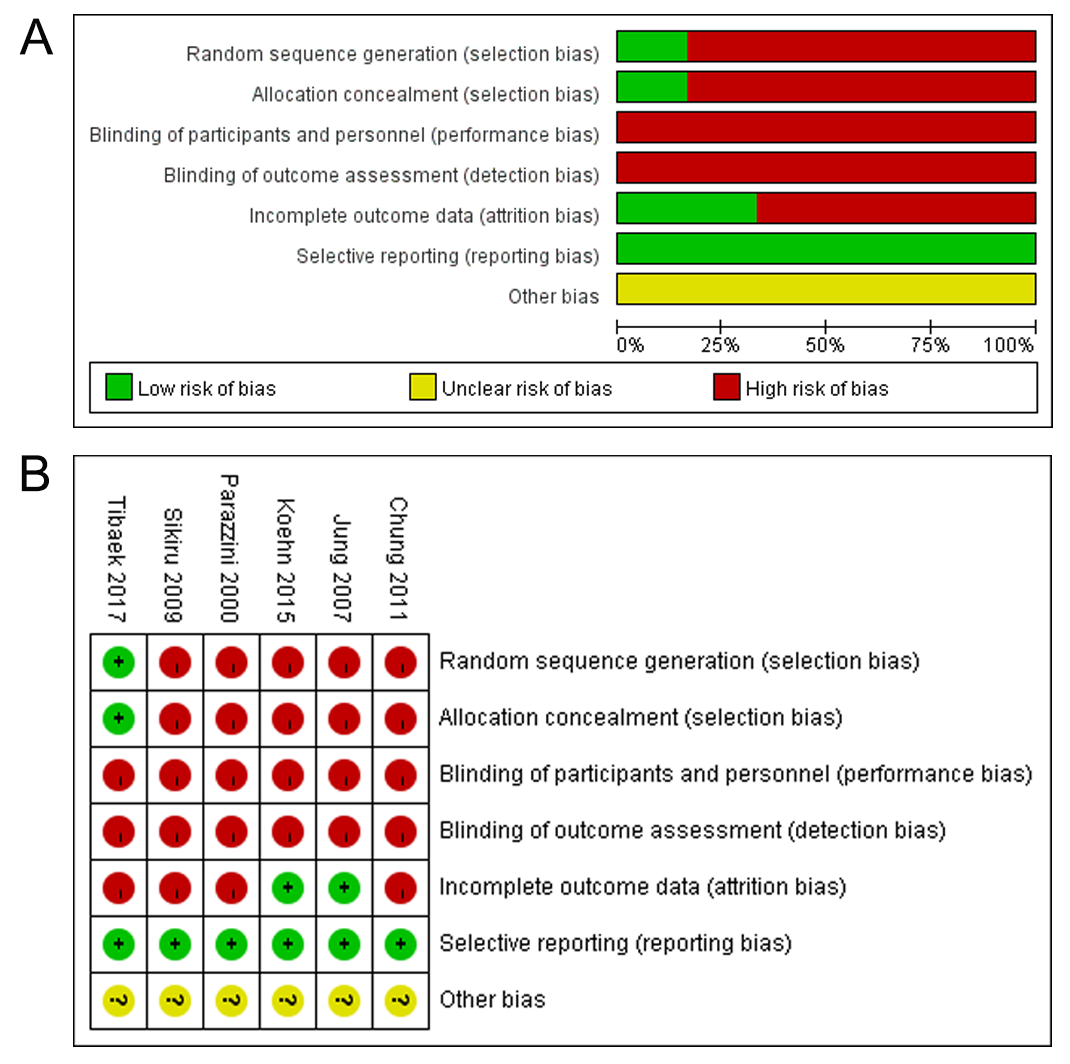

Supplement: Supplementary Figure 1 — Results of risk of bias assessment in each included study: (A) risk of bias graph and (B) risk of bias summary. [file Image_1.TIF]

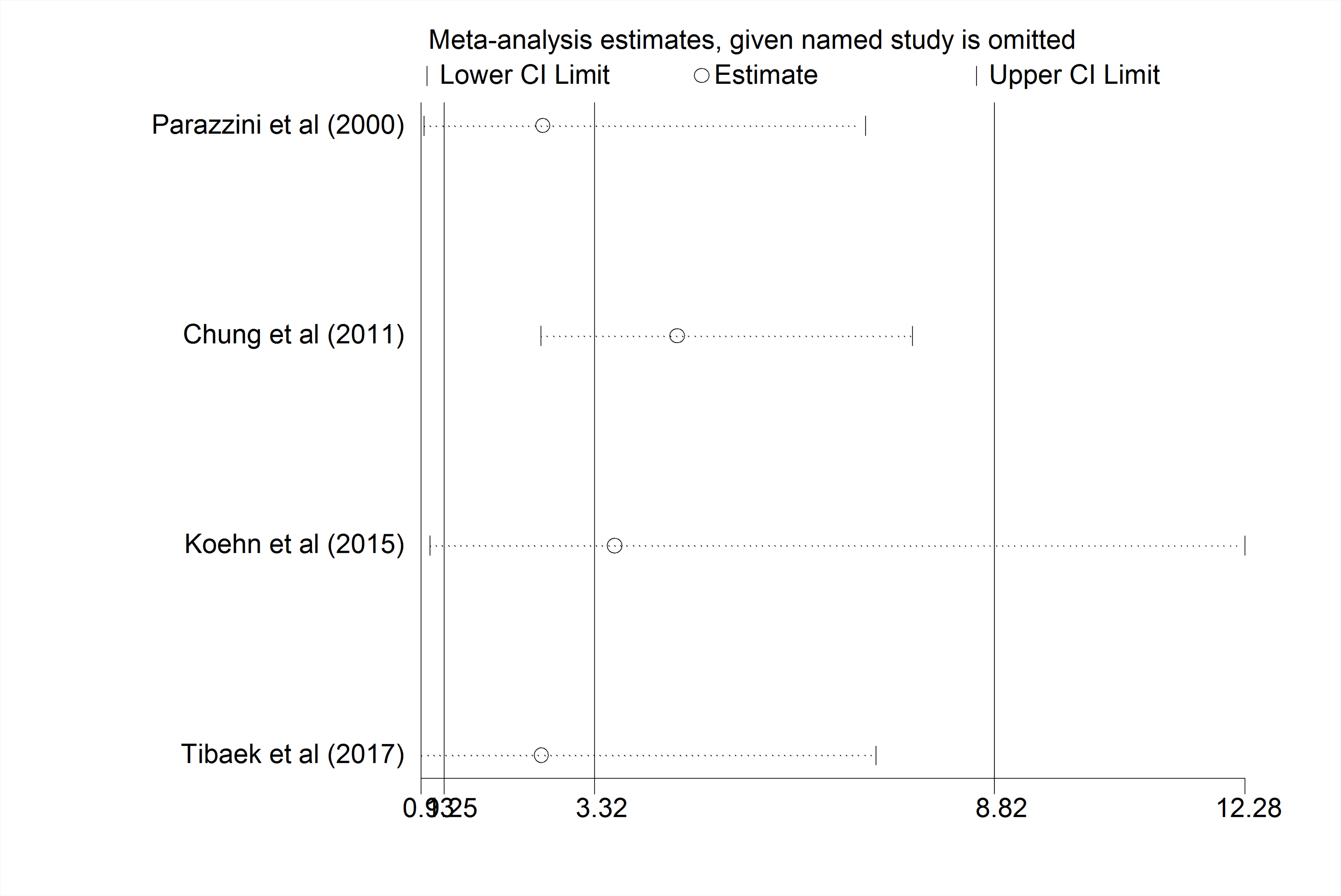

Supplement: Supplementary Figure 2 — Forest plot for sensitivity analysis. [file Image_2.TIF]

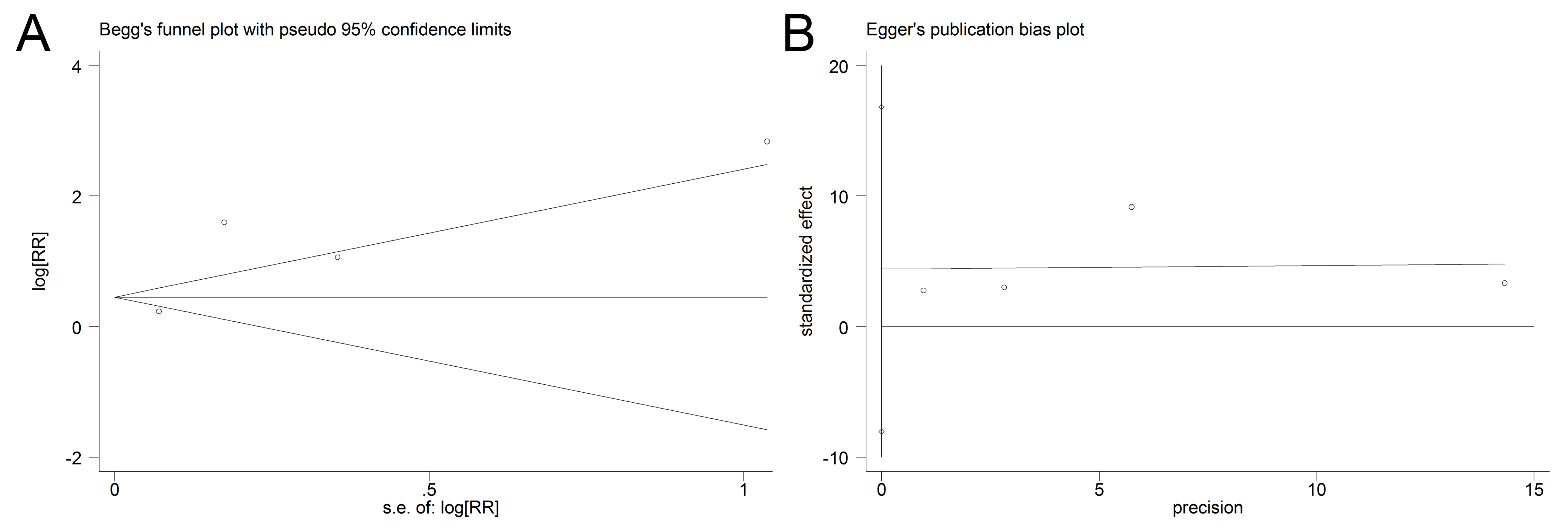

Supplement: Supplementary Figure 3 — Begg's and Egger's tests to detect publication bias. [file Image_3.TIF]
