# Supplementary material for: Significant Increase of Erectile Dysfunction in Men With Post-stroke: A Comprehensive Review
Source: Front Neurol. 2021 Jul 28;12:671738. doi: 10.3389/fneur.2021.671738 (PMC8355431; doi:10.3389/fneur.2021.671738)
Supplement: Supplementary file 5 [file Table_2.DOC]

| Item | Parazzini  2000 | Tibaek 2017 |
| --- | --- | --- |
| 1) Define the source of information (survey, record review) | Yes | Yes |
| 2) List inclusion and exclusion criteria for exposed and unexposed subjects (cases and controls) or refer to previous publications | Yes | Yes |
| 3) Indicate time period used for identifying patients | Yes | Yes |
| 4) Indicate whether or not subjects were consecutive if not population-based | NO | NO |
| 5) Indicate if evaluators of subjective components of study were masked to other aspects of the status of the participants | NO | Yes |
| 6) Describe any assessments undertaken for quality assurance purposes (e.g., test/retest of primary outcome measurements) | Yes | Yes |
| 7) Explain any patient exclusions from analysis | Yes | Yes |
| 8) Describe how confounding was assessed and/or controlled. | NO | Yes |
| 9) If applicable, explain how missing data were handled in the analysis | NO | NO |
| 10) Summarize patient response rates and completeness of data collection | Yes | Yes |
| 11) Clarify what follow-up, if any, was expected and the percentage of patients for which incomplete data or follow-up was obtained | NO | NO |

Supplementary Table 2. The methodological quality of the cross-sectional studies.
