# Supplementary material for: Significant Increase of Erectile Dysfunction in Men With Post-stroke: A Comprehensive Review
Source: Front Neurol. 2021 Jul 28;12:671738. doi: 10.3389/fneur.2021.671738 (PMC8355431; doi:10.3389/fneur.2021.671738)
Supplement: Supplementary file 6 [file Table_3.DOC]

| Study | Selection | | | | Comparability | | Exposure/Outcome | | | Total scores |
| --- | --- | --- | --- | --- | --- | --- | --- | --- | --- | --- |
|  | 1 | 2 | 3 | 4 | 5 | 6 | 7 | 8 | 9 |  |
| Chung et al, 2011 | Yes | Yes | Yes | Yes | Yes | No | Yes | Yes | Yes | 8 |
| Koehn et al, 2015 | Yes | Yes | Yes | Yes | Yes | No | Yes | No | No | 6 |
| Jung et al, 2007 | Yes | Yes | Yes | Yes | Yes | Yes | No | No | No | 6 |
| Sikiru et al, 2009 | Yes | Yes | Yes | Yes | Yes | No | Yes | No | No | 6 |

Supplementary Table 3. Newcastle-Ottawa Scale assessment of the quality of the case-control studies and cohort studies.

NOTE: 1. indicates that the exposed cohort was representative of the population; 2. Indicates that the non-exposed cohort was drawn from the same population; 3. Indicates that the exposure ascertainment was from secure records or a structured interview; 4. Indicates that outcome of interest was not present at start of study; 5. Indicates that the cohorts were comparable for age and sex; 6. Indicates that the cohorts were comparable on all additional factor(s) reported; 7. Indicates that outcome was assessed from a secure record; 8. Indicates that follow-up was long enough for outcomes to occur; 9. Indicates that follow-up was complete.
